# Integrating eFAST in the initial management of stable trauma patients: the end of plain film radiography

**DOI:** 10.1186/s13613-016-0166-0

**Published:** 2016-07-11

**Authors:** Sophie Rym Hamada, Nathalie Delhaye, Sebastien Kerever, Anatole Harrois, Jacques Duranteau

**Affiliations:** Anesthesiology and Critical Care Department, Service de Réanimation chirurgicale, AP-HP, Hôpital Bicêtre, Hôpitaux Universitaires Paris Sud, 78 rue du Général Leclerc, 94275 Le Kremlin Bicêtre, France; Anesthesiology and Critical Care Department, AP-HP, Hôpital Pitié-Salpêtrière, Hôpitaux Universitaires Pitié-Salpêtrière, 47-83 Boulevard de l’Hôpital, 75013 Paris, France; Department of Anesthesiology and Critical Care, Lariboisière University Hospital, AP-HP, Paris, France; ECSTRA Team, Epidemiology and Biostatistics Sorbonne Paris Cité Research Centre, UMR 1153, INSERM, Paris, France; University of Paris VII Denis Diderot, Paris, France

**Keywords:** Severe trauma, Stability, Resuscitation, Imaging, Diagnostic accuracy, Ultrasound

## Abstract

**Background:**

The initial management of a trauma patient is a critical and demanding period. The use of extended focused assessment sonography for trauma (eFAST) has become more prevalent in trauma rooms, raising questions about the real “added value” of chest X-rays (CXRs) and pelvic X-rays (PXR), particularly in haemodynamically stable trauma patients. The aim of this study was to evaluate the effectiveness of a management protocol integrating eFAST and excluding X-rays in stable trauma patients.

**Methods:**

This was a prospective, interventional, single-centre study including all primary blunt trauma patients admitted to the trauma bay with a suspicion of severe trauma. All patients underwent physical examination and eFAST (assessing abdomen, pelvis, pericardium and pleura) before a whole-body CT scan (WBCT). Patients fulfilling all stability criteria at any time in transit from the scene of the accident to the hospital were managed in the trauma bay without chest and PXR.

**Results:**

Amongst 430 patients, 148 fulfilled the stability criteria (stability criteria group) of which 122 (82 %) had no X-rays in the trauma bay. No diagnostic failure with an immediate clinical impact was identified in the stability criteria group (SC group). All cases of pneumothorax requiring chest drainage were identified by eFAST associated with a clinical examination before the WBCT scan in the SC group. The time spent in the trauma bay was significantly shorter for the SC group without X-rays compared to those who received any X-ray (25 [20; 35] vs. 38 [30; 60] min, respectively; *p* < 0.0001). An analysis of the cost and radiation exposure showed savings of 7000 Є and 100 mSv, respectively.

**Conclusions:**

No unrecognized diagnostic with a clinical impact due to the lack of CXR and PXR during the initial management of stable trauma patients was observed. The eFAST associated with physical examination provided the information necessary to safely complete the WBCT scan. It allowed a sensible cost and radiation saving.

## Background

The initial management of a trauma patient is a critical period combining at the same time the need to make a rigorous injury assessment, find sources of bleeding, stabilize vital functions and define a therapeutic strategy. This approach is outlined in the “advanced trauma life support” (ATLS) guidelines. These guidelines propose a complete primary survey that includes diagnostic adjuncts such as chest X-rays (CXRs), pelvic X-rays (PXR) and focused assessment with sonography for trauma (FAST) performed simultaneously alongside initial resuscitation.

Ultrasonography has recently emerged as an essential point-of-care device in the trauma bay [1, 2]. It has become the extension of the practitioner’s hand and his ultrasound stethoscope allowing assessment of the pleura [3–5], brain [6], heart [7] and stomach residual volume [8], as well as acting as a guide to facilitate vascular access [9] or other invasive procedures. The diagnostic performance of pleural ultrasonography is convincing and shows better results compared to CXR, especially in the context of trauma [4, 5], where CXR is performed in the supine position due to the need for spine immobilization. However, the use of the so-called extended FAST (eFAST) [10] procedure has not yet been validated as a standard of care to replace CXR in the initial management of trauma patients. Concerning PXR, there is an increasing amount of evidence demonstrating the poor performance of PXR and their minimal value in decision-making during the primary survey of haemodynamically stable patients [11, 12]. Pelvic CT scans are instead superior in terms of diagnostic accuracy for fractures and active bleeding [13]. Thus, the added value of CXR and PXR can be questioned in haemodynamically stable trauma patients when eFAST is used during the primary survey and a whole-body CT (WBCT) scan in the secondary survey.

The aim of this study was to evaluate the effectiveness of a management protocol integrating eFAST and excluding X-rays in stable trauma patients.

## Patients and methods

This prospective, interventional study was performed in a single academic trauma centre in France (Bicêtre Hospital, Kremlin-Bicêtre, France) between October 2013 and January 2015. All patients with blunt trauma who were admitted directly to the trauma bay were included. The study was approved by the local institutional review board (“Comité de Protection des Personnes”, No. SC 13-024).

### Protocol

The Bicêtre trauma centre is a 1300-bed institution on the southern edge of Paris, which receives an average of 500 trauma patients each year. The French emergency medical system and prehospital care organization have been described previously [14]. All patients admitted in the trauma bay were suspected of severe trauma, according to the French trauma triage criteria [15]. The standard trauma care in the hospital is in concordance with the ATLS protocol and French recommendations of the «Haute Autorité de Santé» [16]. It includes an initial survey with imaging (CXR, PXR and FAST), resuscitation and a WBCT scan for complete injury assessment [17].

**Fig. 1:**
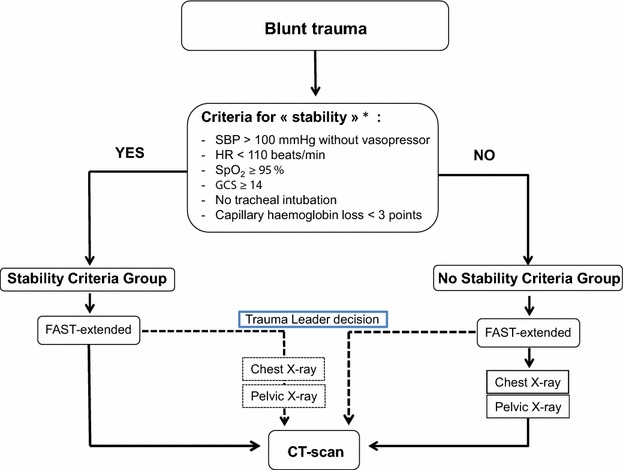
Institutional protocol. *Criteria collected during prehospital and trauma bay period (before CT scan). *SBP* systolic blood pressure, *HR* heart rate, *SpO*_*2*_ peripheral oxygen saturation, *GCS* Glasgow Coma Scale, *FAST* focused assessment with sonography for trauma, *CT scan* computed tomography

To select a population of haemodynamically stable trauma patients, we defined «stability criteria» as: minimum systolic arterial blood pressure (SAP) > 100 mmHg without vasopressor; maximum heart rate (HR) < 110 beats/min; minimum peripheral oxygen saturation (SpO_2_) > 94 %; minimum Glasgow Coma Score (GCS) > 13; no tracheal intubation; and a difference in capillary haemoglobin of <3 points between the first measurement performed by the prehospital team and the second measurement performed upon patient admission. These criteria had to be fulfilled at any time during transport from the scene of the trauma to hospital admission (Fig. 1). Patients were categorized into two groups: those who fulfilled all stability criteria were classed in the stability criteria group (SC group), whilst those who failed to meet one or more of the criteria were classed in the no stability criteria group (NSC group).

Within 5 min of admission to the trauma bay, all patients underwent a physical examination (palpation from hair to toes and thoracic breathing sounds) and an extended ultrasonography (eFAST). Physical examination of the chest was considered positive if any crepitus or abnormal breathing sounds (BS) were present during auscultation. Physical examination of the pelvis was considered positive if there was any instability during compression. The eFAST including the assessment with sonography of abdomen, pelvis, pericardium and pleura [4, 18] was performed by the trauma leader (intensivist). Practitioners of the team had varied experience in echography; nevertheless, all of them had undergone the basic training needed to perform ultrasound examination and had at least 50 supervised eFAST examinations [19].

When patients fulfilled all the stability criteria, no CXR or PXR was performed. However, the trauma leader could request those X-rays if needed, but had to justify his choice by writing down the reason. Any argument showing that the X-ray had, or could have, modified their management strategy was considered as correct justification by the authors. On the other hand, patients in the NSC group were systematically subjected to C-PXR, using a portable device within 10 min of arrival at the trauma bay. These X-rays were analysed by the trauma leader in the trauma bay. The trauma leader was allowed to cancel the X-rays (one or two) if he considered that physical examination and eFAST provided the information needed to guide his strategy. If required, resuscitation was initiated (chest drainage, intubation, fluid load, transfusion, pelvic belt) and the patient was transferred as quickly as possible to the radiology department for a WBCT scan.

The WBCT scan was considered as the “gold standard” for the diagnosis of pneumothorax (PNO), haemothorax (HMO) and pelvic fracture. PNO was considered as substantial if drainage was needed. A diagnostic failure of the procedure was defined as a substantial clinical worsening of a missed injury (drainage of a missed haemo-/pneumothorax, urgent embolization of a fractured pelvis, unpredicted thoracic or pelvic surgery).

### Data collection

Most data were collected in a standardized trauma file, which has existed since 2010 and collects prehospital and initial hospital management information. The following items were recorded: demographic characteristics, injury mechanism, lowest prehospital SAP, highest HR, lowest GCS, lowest SpO_2_, initial capillary haemoglobin, care provided during the prehospital phase (tracheal intubation, vasopressor) and SAP, HR, SpO_2_, capillary haemoglobin and GCS upon arrival at the hospital. Thoracic and pelvic physical examination findings, results of eFAST and X-rays, CT imaging of the chest and pelvis, relevant clinical management in the first 24 h and outcome were also recorded. The following scores were calculated after anatomic and physiological assessments had been completed: Abbreviated Injury Scale (AIS) score, Injury Severity Score (ISS) [20] and Simplified Acute Physiology Score (SAPS II) [21]. Most data were recorded systematically in the prospective local trauma registry named «TraumaBase» (www.traumabase.eu; authorization No. 911,461).

### Imaging cost characteristics

The costs of CXR and PXR were estimated at 28.2 Є and 27.5 Є, respectively. Radiation exposure levels for CXR were estimated at 0.1 and 0.7 mSv for PXR.

### End-points

The primary end-point was the rate of unrecognized diagnosis with a clinical impact due to the lack of CXR and PXR in the protocol integrating eFAST in stable trauma patients. Secondary end-points were: (1) time spent in the trauma bay, (2) reduction in body irradiation and (3) cost savings.

### Statistical analysis

Data are presented as number (percentage) for qualitative variables and median [25th; 75th percentile] for quantitative variables. Fisher’s exact test and Wilcoxon’s nonparametric rank sum test were used to compare these two types of variable, respectively. A historical cohort of patients admitted the year before the implementation of the procedure (September 2012–2013) was used to compare the practices (X-rays performed on 91 % patients, *n* = 228/252). All tests were two-sided with *p* ≤ 0.05 considered statistically significant. Statistical analyses were performed using R 3.1.1 (http://www.R-project.org/) packages.

## Results

**Fig. 2:**
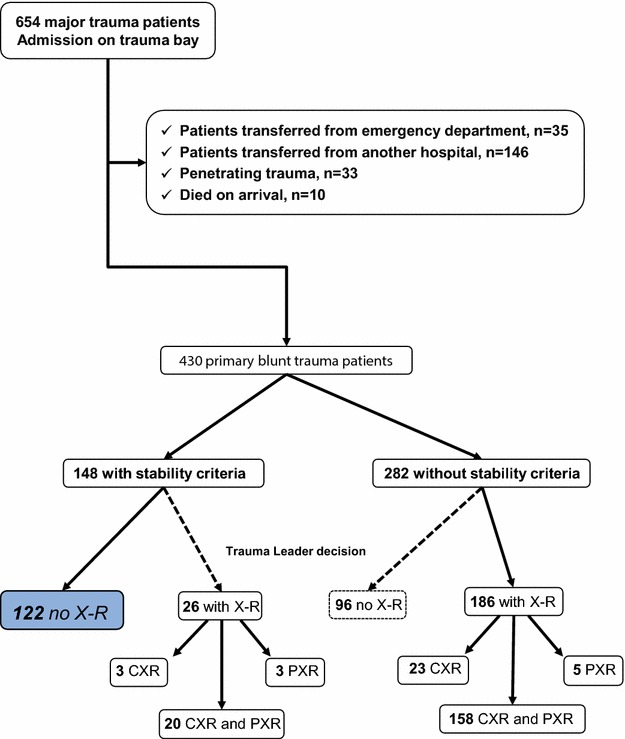
Flow chart of the study. *X-R* X-ray, *CRX* chest X-ray, *PXR* pelvic X-ray

Between October 2013 and January 2015, 654 trauma patients were admitted to our trauma centre, including 430 blunt trauma patients (flow chart is shown in Fig. 2). Of these, 148 fulfilled all the stability criteria (SC group) and 282 did not (NSC group). The demographic and clinical characteristics of the patients are listed in Table 1.

**Table 1:** Demographic and clinical characteristics of the study population

	Stability criteria group (*n* = 148)	No stability criteria group (*n* = 282)
Age (years)	31 [22, 43]	36 [26; 53]
Sex (male) (%)	114 (77)	196 (70)
BMI (kg/m^2^)	24 [22, 26]	24 [22, 28]
ASA	1 [1; 1]	1 [1, 2]
Vittel criteria	1 [1, 2]	3 [2, 4]
SAPS 2	13 [8, 19]	25 [15, 40]
ISS	9 [4, 14]	17 [9, 28]
*Mechanism of injury n (%)*		
MVA	40 (27)	98 (35)
Motorbike	59 (40)	53 (19)
Cyclist	3 (2)	10 (4)
Pedestrian	9 (6)	34 (12)
Fall	32 (22)	66 (23)
Other	5 (3)	21 (7)
Chest AIS > 2	32 (22)	114 (40)
Pelvis AIS > 2	14 (10)	45 (16)
Catheters before CT scan	38 (26)	183 (65)
Mechanical ventilation (days)	1 [0; 1]	1 [0; 5]
Intensive care unit length of stay (days)	2 [2; 5]	5 [3; 12]
Hospital length of stay (days)	8 [4; 16]	13 [7; 23]
Mortality	1 (0.7)	31 (11)

Values shown are n (%) or median [25th; 75th percentile]

*BMI* body mass index, *ASA* American Society of Anaesthesiologists. *SAPS 2* Simplified Acute Physiology Score, *ISS* Injury Severity Score, *MVA* motor vehicle accident, *AIS* Abbreviated Injury Scale

In the SC group, 122 (82 %) patients out of the 148 patients had no CXR and no PXR. The comparison of physical examination, CXR and eFAST for the diagnosis of pneumothorax, pneumothorax requiring chest drainage and haemothorax are listed in Table 2. No tension pneumothorax or massive haemothorax was diagnosed in the SC group, and no unrecognized diagnostic due to the lack of CXR and PXR was observed. The eFAST associated with physical examination allowed the diagnosis of 100 % of pneumothoraces necessitating drainage (*n* = 5) and one haemothorax out of three (but none of the haemothoraces required a chest tube within the first 24 h). The patients had the chest tube inserted mainly after WBCT scan (four out of five pneumothoraces) even if the diagnosis had been made before in the trauma bay. One patient had an unstable pelvic fracture diagnosed clinically but without any haemodynamic implication related to bleeding. He underwent a pelvic angiography with arterial embolization after the WBCT scan. No emergency thoracotomy or thoracic arterial embolization was required in the SC group.

**Table 2:** Comparison of physical examination, chest X-ray (CXR) and e-FAST for the diagnosis of pneumothorax, pneumothorax requiring chest drainage and haemothorax in the stability criteria group

	CT diagnosis (*n* = 148)	eFAST (*n* = 148)	Physical examination (*n* = 148)	Chest X-ray (*n* = 23)
All pneumothoraces	21	6 (29 %)	4 (19 %)	3 (14 %)
Drained pneumothoraces	5	5 (100 %)	3 (60 %)	3 (60 %)
All haemothoraces^a^	3	1 (33 %)	1 (33 %)	0 (0 %)

^a^None of the haemothoraces required a chest drainage within the first 24 h

X-rays were performed in 26 patients (18 %) in the SC group. The trauma leaders justified these X-rays as follows: (1) for five patients with a haemothorax and/or pneumothorax diagnosed by eFAST and three patients with a suspected pelvic fracture, the physician wanted radiographic confirmation; (2) one patient had a PXR to check for a symphyseal diastasis before urgent urinary catheterization for acute urinary retention recognized by eFAST; (3) one patient had a drop in capillary haemoglobin; (4) one patient became unstable after tracheal intubation for pain relief; (5) in 10/26 patients, the physician in charge of the patient forgot about the protocol; and (6) in five patients, non-compliance with the protocol was not justified. In 23 patients where CXR was performed (out of protocol), no additional diagnostic information was established.

In the SC group, there was a saving of 250 X-rays (125 CXR and 125 PXR) leading to a mean radiation avoidance of 100 mSv. In the NSC group, the mean radiation avoidance was 93 mSv with 101 CXR and 119 PXR saved. The estimated cost saving was 7000 € in the SC group and 6000 € in the NSC group.

A comparison of the time spent in the trauma bay within the SC group showed that patients who had no X-rays spent on average 25 min [20; 35] in the trauma bay versus 38 min [30; 60] for those who had at least one X-ray (*p* < 0.0001), with no statistical difference in their Injury Severity Score (*p* = 0.8).

Concerning the global trend of imaging in the trauma bay, the prescription of X-rays also decreased in the NSC group during the study. Indeed, the rate of any X-ray prescription in the NSC group was 66 % (*n* = 186/282) during the study period compared to 91 % (*n* = 228/252) during the preceding period (historical cohort, September 2012–2013) (*p* < 0.0001).

## Discussion

This is the first study to analyse the effectiveness of a protocol integrating eFAST and excluding all X-rays during the primary survey in stable trauma patients. The absence of X-rays did not lead to any undiagnosed injury that could be life threatening.

Our cohort of trauma patients is comparable to other cohorts of multiple injured patients in the literature (i.e. mostly young men with no medical history) [22]. The distribution of ISS showed a wide range of injury severities in our patients, who were triaged by a physician on-scene using an algorithm [14]. Thus, organizing a “fast-track” management without CXR and PXR for the most haemodynamically stable appeared to be safe [2, 5].

Protocol compliance was observed in 82 % of the patients in the SC group. Protocol deviations were mostly due to oversight by the trauma team leader and/or to usual practice (34 %). Apart from these, the authors considered the practitioners’ justifications as “right” in only 31 % of cases. The remaining justifications were considered to be due to the trauma leaders’ unfamiliarity with a new modality and a substantial change to a well-established protocol [23]. Indeed, a normal CXR and PXR are reassuring before allowing a patient to leave the trauma bay for the radiology department. This is especially true for practitioners who do not feel comfortable performing eFAST.

The initial assessment and management of injured patients have evolved with the application of advanced imaging techniques. The role of plain film radiography is becoming increasingly less important in the initial evaluation of trauma patients. This change is based on evidence that plain film radiography adds little decision-making information in haemodynamically stable patients with negative physical examination of the neck or pelvis [24–26]. Routine X-rays for all patients in the trauma resuscitation room, although not clearly mandated by the early versions of ATLS, have always been a strong recommendation. However, the growing role of ultrasound in the trauma bay and WBCT scan has changed the daily practice with no update in the guidelines [27, 28]. WBCT scans have become the gold standard for the diagnosis of solid organ [29], retroperitoneal [30] and orthopaedic injuries to the pelvis and spine [31, 32]. In our practice, once a patient is admitted to the trauma bay, whatever their haemodynamic status, the protocol will direct them for a WBCT scan for an exhaustive injury assessment, before or after an emergency haemostatic intervention (surgery or interventional radiology).

For stable patients, the results of our study show that a combination of eFAST and physical examination can rule out a potential life-threatening injury and safely direct the patient for a WBCT scan. Our analysis of the performance of eFAST for relevant pneumothoraces shows that this procedure is suitable for daily use in the trauma room and appears sufficient to manage the patients safely (100 % sensitivity for clinically relevant pneumothoraces; Table 2). The poor overall performance of ultrasound to diagnose pneumothorax in our study (sensitivity 27 %) compared to other published literature (43–92 %) [4, 5] can be explained by two points. Firstly, the sensitivity is assessed in a selected cohort of ultra-stable trauma patients with a very low ISS (9 [4–14]). The pneumothoraces considered on the CT scan as non-clinically relevant (16 out of 21; 15 not diagnosed by eFAST) were small to tiny (sometimes just a bubble) and did not even necessitate drainage. A consensual method to quantify pneumothorax would be needed to properly compare the performance studies using CT scan as the gold standard. Secondly, these differences could be due to the individual competence of the practitioners performing ultrasound in our centre. Ultrasound examination is known to be operator dependent, and although all trauma practitioners receive training prior to joining the trauma team, experience between individuals may vary. In our centre, 36 % of the practitioners were junior doctors with less extensive experience in ultrasound. Moreover, beyond the poor performance of ultrasound to diagnose non-clinically relevant pneumothorax in this category of patients, CXR has already been shown to have poor sensitivity and specificity in detecting thoracic injury in haemodynamically stable blunt trauma patients [33].

For PXR done in the SC group (protocol deviation), the diagnosis of a fracture in two patients did not lead to any change in their management (no pelvic binding, no angiography). Currently, the following scenarios should lead to a PXR in the trauma room: a haemodynamically unstable patient and/or patient who is transferred for an urgent procedure in order to identify a pelvic fracture that could be a source of haemorrhage and the suspicion of hip dislocation in order to relocate the bone before a WBCT scan [11].

In stable patients, before the present protocol, we used to wait for the development of the CXR and PXR, even though we knew that these X-rays were almost certainly going to be negative. Thus, the X-ray technicians were unnecessarily busy without contributing towards the necessary decision-making information. Currently, we immediately dismiss the radiology technician (a member of the trauma team) and transfer the patient earlier for a WBCT scan. The comparison of the time spent in the trauma bay between patients of the SC group managed according to the procedure versus those who underwent at least one X-ray showed a median saving of 13 min, with no difference in the severity of their injuries. Being faster might have no impact on the outcome of stable trauma patients, but it is known that any delay in treatment of unstable patients in haemorrhagic shock can increase their mortality [34, 35]. Thus, a faster processing of stable trauma patients will result in a more fluid system freeing the trauma room sooner and allowing it to receive another patient that might benefit from immediate acute care. Moreover, in 80 % of the cases of identified pneumothoraces in the SC group, patients received the chest tube after the WBCT scan. This decision by the trauma leader was based on the fact that there was no respiratory failure, and therefore, gaining a clear idea of the state of the lung before drainage was the more secure option. Indeed, the knowledge of part of the underlying injuries identified on eFAST gives the trauma leader a great advantage should the clinical situation state of the patient worsen. Finally, increasing the speed of transfer to a WBCT also allowed the quicker release of the surgical team on standby for the CT injury assessment.

Radiation exposure is a major concern in the diagnostic strategy as it may induce potential adverse effects [36, 37]. Nevertheless, evidence for the use of WBCT scans in trauma patients is now strongly convincing as this procedure has been proven to reduce mortality [27, 38, 39]. CT scanning technologies have improved over the past decade, reducing the effective dose of radiation to 5–10 mSv. Although the amount of radiation in CXR and PXR represents only 8–15 % of the amount in a WBCT scan, the total radiation dose throughout the hospital stay would be reduced by eliminating pointless examinations. Furthermore, although outcome is still the major concern for the practitioner, cost is an ever-growing consideration in medicine. If this practice were to become widespread throughout France, there would be considerable savings to the healthcare system.

The major problem of a management algorithm lies in the definition of haemodynamic instability, which is imprecise and variable. A SAP < 90 mmHg is usually the major criterion to define instability. Nevertheless, the cut-off is sometimes different (SAP < 100 mmHg) [40] or sometimes combined with resuscitation parameters such as 2000-ml volume load or two red blood cell concentrates [41], or sometimes even vasopressors [42]. Some authors have proposed a combination of physiological variables such as SAP > 100 mmHg and Sp0_2_ > 90 % [43] or SAP > 90 mmHg and HR < 100 bpm [39] to limit the use of CXR in trauma patients. In our study, the stability criteria were more restrictive and precise. Practitioners have evolved from a systematic model based on old recommendations [17] towards a more reasoned model that integrates and relies upon data from eFAST. After evaluation of the procedure, our practitioners became confident with eFAST and cancelled X-rays for patients who would normally receive them as part of standard care (as shown by the global trend of a decrease in the use of imaging seen in the NSC group).

 Our study has several limitations. First, this is a one centre, observational study and the results should be interpreted in this context. Secondly, the practice of eFAST requires training [40] and is operator dependent. In our centre, no radiologist is usually present on the trauma bay and the trauma leader performs the eFAST as exhaustively and quickly as possible, focussing particularly on clinical abnormalities. The good performance in detecting clinically relevant pneumothoraces, but the relatively low detection ability for overall pneumothorax (including small and tiny), already addressed in the discussion, can also be seen as a limitation of our study. Nevertheless, this does not change the primary outcome result, which concludes that CXR and PXR are not useful in stable patients and so lead to unnecessary time in the trauma room. Finally, the results of this study should be analysed with caution and require further evaluation. Indeed, the study has been performed in a high-volume trauma centre, receiving an average of 500 “suspected severe trauma patients” per year. The organization and skills of the trauma team members in this practice may not be comparable with those in some small emergency departments. Indeed, the organization of our trauma centre allows patients to have their WBCT scan within the first 15–30 min of arrival so results might not be generalizable to all centres. This study represents the first step towards streamlining trauma management and should be followed by testing the utility of CXR and PXR in all trauma patients.

## Conclusion

 The evaluation of our protocol integrating eFAST and excluding X-rays in stable trauma patients showed no unrecognized diagnostic with a clinical impact due to the lack of CXR and PXR. The eFAST associated with physical examination provided the information necessary to safely and quickly complete the WBCT scan. This study represents the first step towards streamlining trauma management and should be followed by testing the utility of CXR and PXR in all trauma patients.

## Abbreviations

CXR: chest X-ray
PXR: pelvic X-ray
FAST: focused assessment with sonography for trauma
eFAST: extended focused assessment with sonography for trauma
CT: computed tomography
WBCT: whole-body computed tomography
SAP: systolic arterial pressure
HR: heart rate
ATLS: advanced trauma life support
GCS: Glasgow Coma Scale
BMI: body mass index
ASA: American Society of Anaesthesiologists
SAPS 2: Simplified Acute Physiology Score
ISS: Injury Severity Score
MVA: motor vehicle accident
AIS: Abbreviated Injury Scale
